# Discovery of Novel Stimulators of Pax7 and/or MyoD: Enhancing the Efficacy of Cultured Meat Production through Culture Media Enrichment

**DOI:** 10.3390/bios14010024

**Published:** 2023-12-30

**Authors:** In-Sun Yu, Yae Rim Choi, Jungseok Choi, Mina K. Kim, Chang Hwa Jung, Min Young Um, Min Jung Kim

**Affiliations:** 1 Division of Food Functionality Research, Korea Food Research Institute, Wanju-gun 55365, Republic of Korea; dlstjs89@gmail.com (I.-S.Y.); uiu7896@gmail.com (Y.R.C.); chjung@kfri.re.kr (C.H.J.); myum@kfri.re.kr (M.Y.U.); 2 Department of Food Science and Human Nutrition and K-Food Research Center, Jeonbuk National University, Jeonju-si 54896, Republic of Korea; minakim@jbnu.ac.kr; 3 Department of Food Science and Engineering, Ewha Womans University, Seoul 03760, Republic of Korea; 4 Department of Animal Science, Chungbuk National University, Cheongju-si 28644, Republic of Korea; jchoi@chungbuk.ac.kr

**Keywords:** Pax7, MyoD, Hanwoo satellite cell, myogenesis, SPR, screening

## Abstract

The principles of myogenesis play crucial roles in the production of cultured meat, and identifying protein stimulators associated with myogenesis holds great potential to enhance the efficiency of this process. In this study, we used surface plasmon resonance (SPR)-based screening of a natural product library to discover ligands for Pax7 and MyoD, key regulators of satellite cells (SCs), and performed cell-based assays on Hanwoo SCs (HWSCs) to identify substances that promote cell proliferation and/or differentiation. Through an SPR analysis, we found that six chemicals, including one Pax7+/MyoD− chemical, four Pax7+/MyoD+ chemicals, and one Pax7−/MyoD+ chemical, bound to Pax7 and/or MyoD proteins. Among four Pax7+/MyoD+ chemicals, parthenolide (0.5 and 1 µM) and rutin (100 and 200 µM) stimulated cell proliferation in the medium with 10% FBS similar to the medium with 20% FBS, without affecting differentiation. Adenosine, a Pax7−/MyoD+ chemical, accelerated differentiation. These chemicals could be potential additives to reduce the reliance of FBS required for HWSC proliferation and differentiation in cultured meat production.

## 1. Introduction

Cultured meat is an innovative and sustainable protein production solution that offers a promising response to the pressing challenges of food security and environmental resilience in the face of a rapidly growing global population and climate change. By cultivating animal-muscle-derived stem cells in a laboratory setting, cultured meat has the potential to revolutionize traditional agriculture and eliminate the need for animal slaughter [1]. However, significant hurdles exist in the development of cultured meat, one of which is the reliance to fetal bovine serum (FBS) as a component of the cell culture medium. FBS is a costly component that significantly increases the production costs of cultured meats and can introduce inconsistencies and uncertainties in the cultured meat production process by including undefined and variable components. To overcome this hurdle, various studies are under way to identify suitable substitutes for FBS that can support cell proliferation and differentiation while maintaining the desired characteristics of cultured meat [2].

To discover alternatives or additives for FBS, a rapid method to screen a large number of substances with appropriate target proteins is required. Surface plasmon resonance (SPR) is a representative and powerful technique for discovering protein–ligand interactions and can be utilized to explore alternative ingredients to replace FBS in cultured meat production. SPR offers label-free detection, the real-time monitoring of interaction kinetics, affinity and specificity of the biomolecular interaction, the quantification of molecular interactions, high sensitivity, and high-throughput screening with small sample requirements [3,4]. SPR, which is used in food safety evaluation, new drug development, diagnosis, and environmental and basic research [5,6], has also been used in protein–protein interaction studies related to myogenesis [7,8]. Furthermore, specific interactions between enolase isoforms and tubulin were observed during muscle stem cell differentiation through SPR analysis [9]. However, SPR alone cannot determine the agonist or antagonist nature of ligands, so additional cellular experiments are required to evaluate the effectiveness of the ligand [10].

Satellite cells (SCs) are primarily utilized in the production of cultured meat, and the regulation of SC proliferation and differentiation is controlled by various transcription factors, including paired box 7 (Pax7) and myoblast determination protein 1 (MyoD). Pax7 is necessary for the maintenance of the quiescent state of muscle SCs but is downregulated during myoblast proliferation [11]. Conditional Pax7 deletion in SCs, primary myoblast, and adult mice strongly arrests cell growth, impairs the regenerative capacity, and downregulates myogenic regulatory factors, limiting regeneration [12,13]. On the other hand, MyoD plays a critical role in initiating and progressing myoblast differentiation into myotubes [14]. MyoD is expressed in myoblast, stimulates transcriptional genes for myoblast differentiation, and is downregulated during differentiation into the myocyte. Primary MyoD−/− cells delay differentiation-specific biomarker expression in a differentiation medium [15]. Both Pax7 and MyoD are important regulators of myogenesis and work together to ensure proper muscle development and regeneration [16,17]. Therefore, these two proteins are good targets for finding substances that promote the proliferation and differentiation of SCs [4,5,6,9,18].

This study aims to discover novel activators of Pax7 and/or MyoD by monitoring ligand–protein interactions using natural compound libraries and the SPR technique. The substances that responded to SPR were categorized into three groups and evaluated for their effects on Hanwoo satellite cell (HWSC) proliferation and differentiation. Hanwoo is a unique small cattle breed native to Korea, renowned for its distinctive flavor, texture, and nutritional value, making it a premium meat source. It plays a significant role in traditional Korean food culture. Therefore, in this study, we chose Hanwoo satellite cells from a variety of satellite cells. The results of this study could contribute to the development of cultured meat production by promoting cell proliferation and differentiation.

## 2. Materials and Methods

### 2.1. Cell Culture and Differentiation

HWSCs were isolated from the buttocks of 30-month-old Hanwoo steers as described in a previous study [19]. In brief, live cells were confirmed through staining with Hoechst 33342 (Thermo Fisher Scientific, Waltham, MA, USA). The isolated cells were then further characterized using FITC anti-sheep CD45 (Bio-Rad, Hercules, CA, USA), PE-CyTM7 anti-human CD56 (BD Biosciences, San Jose, CA, USA), FITC anti-sheep CD31 (Bio-Rad, Hercules, CA, USA), and APC anti-human CD29 antibody (Biolegend, London, UK). The stained cells underwent purification via the SH800S cell sorter (SH800S, Sony, Tokyo, Japan). Ultimately, the CD45−CD31−CD29+CD56+ cell population was obtained. The cell culture dishes were coated with 5 μg/cm^2^ collagen I, rat tail (Corning, NY, USA). HWSCs were cultured in a growth medium (GM) composed of Ham’s F-10 nutrient mix (F-10; Gibco, Waltham, MA, USA), 20% FBS (Gibco), and 1% penicillin/streptomycin (P/S; Gibco) and differentiated in a differentiation medium (DM; F-10 + 2% FBS + 1% P/S) at 37 °C in an atmosphere of 5% CO_2_.

### 2.2. Surface Plasmon Resonance (SPR)

**Figure 1:**
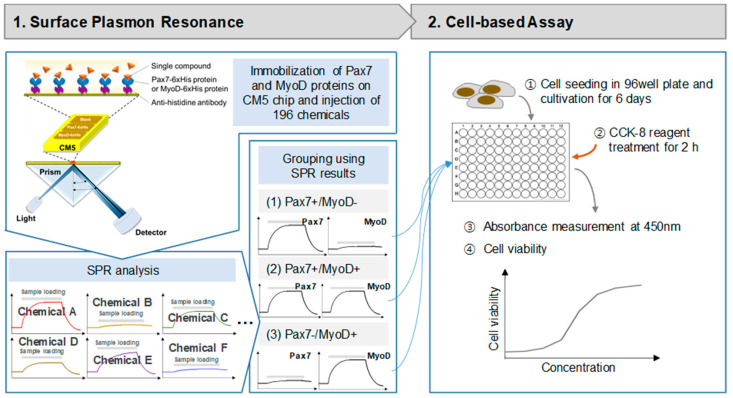
Schematic diagram of surface plasmon resonance (SPR) system and cell-based assay.

The SPR experiment for discovering chemicals binding to the selected proteins was performed using a Biacore T200 (GE healthcare, Chicago, IL, USA) at 25 °C. The SPR experiment process is depicted in Figure 1. Pax7-6xHis and MyoD-6xHis (Proteintech, Wuhan, China) were immobilized on a CM5 sensor chip using a standard amine coupling kit (GE healthcare) with HBS-EP as a running buffer. The CM5 chip was sequentially treated with EDC and NHS (1:1, *v*/*v*) for 10 min, anti-histidine antibody (GE healthcare) for 10 min, and ethanolamine for 7 min at a flow rate of 10 μL/min. Then, Pax7-6xHis and MyoD-6xHis (40 μg/mL) were injected on two flow cells (FC2, F3). FC1 was activated simply by using a standard amine coupling procedure as a reference. Then, the binding properties of proteins to 196 chemicals (Selleck Chemicals L1400, Houston, TX, USA) were measured (Supplementary Table S1). Each chemical was used by diluting 1/1000 in HSB-EP buffer from the stock solution of the Selleck Natural Product Library (purity > 95%) dissolved in DMSO before use. Chemicals were injected to FCs for 60 s at a flow rate of 30 μL/min, followed by dissociation for 120 s. An amount of 0.1% DMSO in HBS-EP buffer was used as a running buffer. All binding affinities were analyzed as the difference between the signals in the protein (FC2 and FC3) and the reference signal (FC1) to exclude non-specific binding. The calculation formula is FC3 (Pax7)-FC1 (blank) or FC2 (MyoD)-FC1 (blank). Each protein binding to a ligand was recorded as a positive value, while non-specific binding to the matrix or blank resulted in a negative value. The noise level was measured to be approximately 0.33 RU, and when the signal-to-noise level was set to 3, the signal was found to be over 1. Considering the noise level in the signal and the response unit (RU) of FC1, only the RU values higher than 1 between 83.10 and 130.93 s were considered signals.

### 2.3. Cell Proliferation and Cytotoxicity

Cell proliferation was measured using a CCK-8 assay using only Pax7+/MyoD− and Pax7+/MyoD+ chemicals (Figure 1). HWSCs (3 × 10^3^ cells/well) in GM with 20% FBS (GM20) were seeded into a 96-well plate. After 24 h, cells were treated with selected chemicals in GM with 10% FBS. The chemicals were treated with selected chemicals in GM with 10% FBS (GM10) every 2 days and cultured for a total of 6 days. At 6 days, CCK-8 solution was added to the cells, and the absorbance was measured at 450 nm using a microplate reader (SpectraMax M2e, Molecular Devices, Sunnyvale, CA, USA). Cytotoxicity was measured using a CCK-8 assay using only Pax7−/MyoD+ chemicals. HWSCs in 96-well plates were grown on GM20 for 6 days and treated with chemicals in DM for 2 days. Then, CCK-8 assay was performed. HWSCs grown in GM20 and GM10 were used as the controls. Cell proliferation rate and cytotoxicity were normalized to GM20 and DM, respectively.

### 2.4. Immunofluorescence

After proliferation for 6 days and differentiation for 4 days, the cells were fixed in 4% paraformaldehyde (Biosesang, Seongnam, Republic of Korea) for 10 min and treated with blocking solution (2% goat serum + 2% horse serum + 0.3% triton X-100 + 1X PBS) for 1 h. Then, the cells were treated with MyoD antibody (1:100, Thermo Fisher Scientific) and MyoG antibody (1:100, Thermo Fisher Scientific) at 4 °C overnight. After washing, cells were incubated with secondary goat anti-mouse lgG (1:500, Thermo Fisher Scientific) and secondary goat anti-rabbit IgG (1:500, Thermo Fisher Scientific) for 1 h at room temperature. Nucleus staining was performed using Hoechest 33342 (Thermo Fisher Scientific). Cell images were measured using Zeiss Axio Imager Z2 (Carl Zeiss, Jena, Germany) under a 10× objective lens.

### 2.5. Statistical Analysis

All statistical analyses were performed using GraphPad Prism 9. The data are represented as the mean ± standard deviation, and comparison was performed using one-way analysis of variance. Differences from GM20 were indicated with an asterisk (red), and differences from GM10 were indicated with a sharp mark (blue). Depending on the number of markers, the *p* values were less than 0.05, 0.01, and 0.001, respectively.

## 3. Results

### 3.1. Characterization of a Chemical That Specifically Binds to Pax7

**Figure 2:**
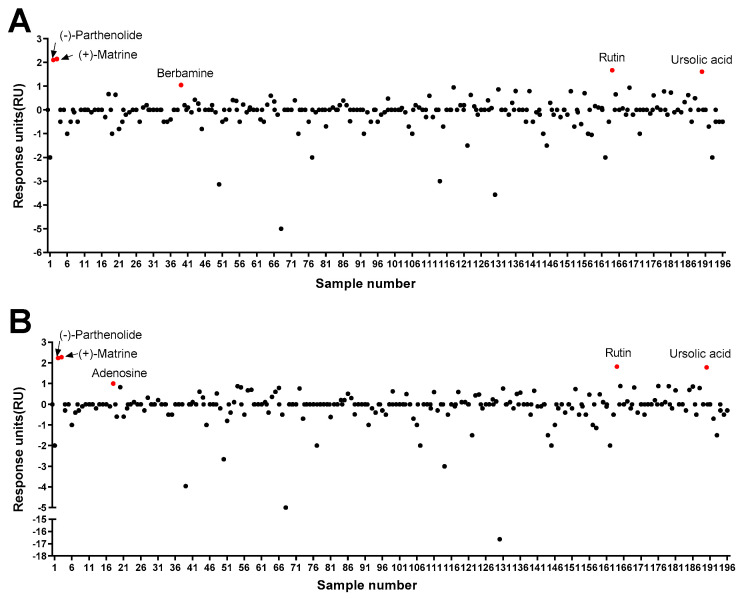
Screening of 196 chemicals for ligand activity to identify chemicals that bind to Pax7 (**A**) and MyoD (**B**) using SPR.

**Figure 3:**
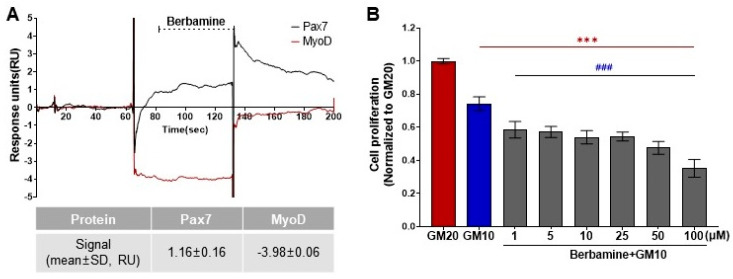
Effects of Pax7+/MyoD- chemicals on SPR-based assay and cell-based assay. (**A**) SPR sensorgram of berbamine for Pax7 and MyoD proteins; (**B**) changes in HWSC proliferation by berbamine treatment at different concentrations using CCK-8 assay. Red (***) indicates significant difference from GM20, and blue (^###^) indicates significant difference from GM10.

As a result of the protein–ligand binding assay using the SPR system, berbamine was the only chemical among 196 chemicals that responded to Pax7, and not MyoD (Pax7+/MyoD−) (Figure 2, Table S1). The SPR response of berbamine to Pax7 and MyoD proteins is shown in Figure 3A. The ΔRU values of berbamine at 147 μM were 1.16 ± 0.16 and −3.98 ± 0.06 RU for Pax7 and MyoD, respectively. To determine the effect of Pax7+/MyoD− chemicals on cell proliferation, HWSCs were treated with berbamine at various concentration ranges under GM10, and the cell proliferation was monitored (Figure 3B). In the control, reducing the FBS content by 50% from GM20 to GM10 decreased the cell proliferation rate to 74.00 ± 0.04%. Berbamine treatment in GM10 reduced HWSC proliferation in a concentration-dependent manner (1–100 μM). The cell-based assays showed that Pax7+/MyoD− chemicals reduced the HWSC proliferation rate over GM10.

### 3.2. Characterization of Pax7+/MyoD+ Chemicals

**Figure 4:**
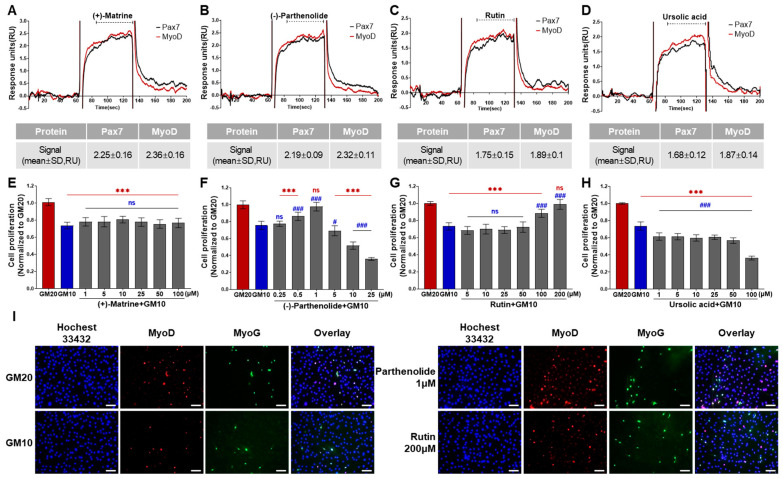
Effects of Pax7+/MyoD+ chemicals on SPR-based assay and cell-based assay. (**A**–**D**) SPR sensorgrams of Pax7+/MyoD+ chemicals for Pax7 and MyoD proteins; (**E**–**H**) changes in HWSC proliferation by Pax7+/MyoD+ chemicals; (**I**) representative fluorescence images of differentiating HWSCs after treatment of parthenolide or rutin during proliferation. Red (***) and blue (^###^) indicate significant difference from GM20 and GM10, respectively. Every scale bar = 100 µm.

Among 196 chemicals, the Pax7+/MyoD+ chemicals were (+)-matrine, (−)-parthenolide, rutin, and ursolic acid (Figure 2, Table S1, and Figure 4A–D). The Pax7+/MyoD+ chemicals had higher ΔRU values than the Pax7+/MyoD− chemicals, and each chemical showed similar ΔRU values for the Pax7 and MyoD proteins. The SPR responses to Pax7 and MyoD were 2.25 ± 0.16 and 2.36 ± 0.16 RU for (+)-matrine (197 μM), 2.19 ± 0.09 and 2.32 ± 0.11 RU for (−)-parthenolide (197 μM), 1.75 ± 0.15 and 1.89 ± 0.1 RU for rutin (164 μM), and 1.68 ± 0.12 and 1.87 ± 0.14 RU for ursolic acid (199 μM), respectively. Then, the effects of the four Pax7+/MyoD+ chemicals on HWSC proliferation were examined using a cell-based assay (Figure 4E–H). By changing the GM20 to GM10, the HWSC proliferation decreased to 74.00 ± 0.04%. The matrine-treated HWSCs had no significant difference in the growth rate with GM10 at all concentrations (1–100 μM). (−)-Parthenolide promoted cell proliferation with increasing concentrations up to 0.5–1 μM, but inhibited cell proliferation with increasing concentrations in the range of 5–25 μM. In particular, the cell proliferation rate at 1 μM if parthenolide was significantly different from GM10, but not significantly different from GM20. The treatment with rutin up to ~50 μM did not affect the growth rate of HWSCs but effectively increased the concentration range from 100 to 200 μM compared to GM10. Particularly, the cell proliferation of the HWSCs treated with 200 μM of rutin was not significantly different from GM20. On the other hand, ursolic acid suppressed HWSC proliferation in a dose-dependent manner (1–100 μM) compared to GM10. Finally, the effects of parthenolide and rutin on differentiation was observed by immunofluorescence using two biomarkers of muscle cell differentiation, MyoD and MyoG (Figure 4I). The expressions of MyoD and MyoG in HWSCs treated with 1 μM of parthenolide or 200 μM of rutin was higher than those in GM10 and GM20. As a result, both parthenolide and rutin promoted myoblast proliferation and did not negatively affect myocyte differentiation.

### 3.3. Effects of Pax7−/MyoD+ Chemicals on HWSC Differentiation

**Figure 5:**
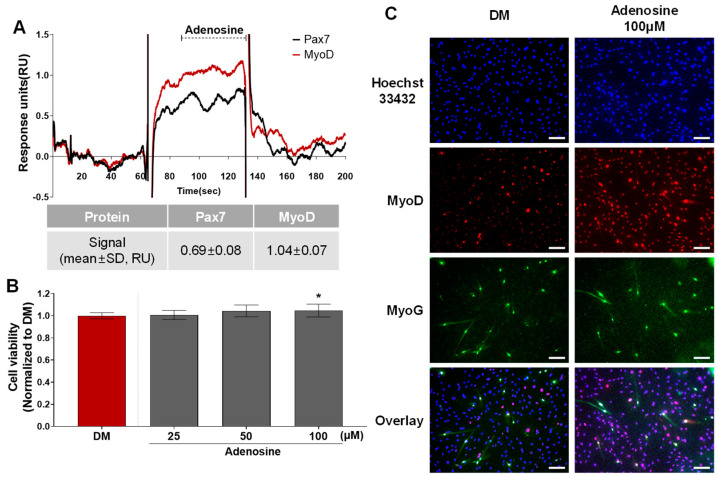
Changes in HWSC differentiation by Pax7−/MyoD+ chemicals. (**A**) SPR sensorgram of adenosine for Pax7 and MyoD proteins; (**B**) cytotoxicity of adenosine; (**C**) immunocytochemical detection of myogenic markers in HWSCs treated with adenosine during differentiation period. * *p* < 0.05 compared to DM. Every scale bar = 100 µm.

According to the protein–ligand binding assay, adenosine is only bound to MyoD, and not Pax7 (Pax7−/MyoD+ chemical) (Figure 2, Table S1). As shown in Figure 5A, the SPR responses for adenosine (45 μM) to Pax7 and MyoD were 0.69 ± 0.08 and 1.04 ± 0.07 RU, respectively. Prior to evaluating the effect of the Pax7-/MyoD+ chemical on HWSC differentiation, non-toxic concentrations were selected through a cell viability assay (Figure 5B). As a result of treating the Pax7-/MyoD+ chemical for 2 days, adenosine had no cytotoxicity up to 100 μM compared to the DM. Therefore, 100 μM of adenosine was selected and treated to the HWSCs during the differentiation period for 7 days (Figure 5C). The expressions of MyoD and MyoG in HWSCs treated with adenosine increased compared to the control (DM). Therefore, adenosine effectively promoted the differentiation of HWSCs as a Pax7-/MyoD+ compound.

## 4. Discussion

The edibility and price reduction of culture media for cultured meat production are common goals of researchers seeking to commercialize cultured meat. Therefore, nutrients such as proteins, carbohydrates, fats, vitamins, and minerals in the culture medium are converted into edible products. There are also studies using serum-free media to replace FBS, which accounts for about 20%. However, serum-free media can delay cell growth, so a serum supply should be maintained for better results. Finding a substitute for serum is also challenging. Therefore, in this study, rather than replacing the existing medium, we attempted to shorten the proliferation time by promoting cell growth and sought to find substances that promote cell growth in ingredients that are not included in the medium but are present in food.

In this study, we employed SPR for the high-throughput screening of protein–chemical interactions, utilizing Pax7 and MyoD proteins as specific biomarkers for proliferation and differentiation in muscle SCs, which are key components of cultured meat. SPR is widely used in the food industry, primarily for critical applications in food safety and quality control. Its application is crucial for detecting food allergens, addressing the increasing global health concerns posed by food allergies, and ensuring the absence of harmful substances in food products [20]. The food industry also has an increasing demand for pathogen detection systems that are sensitive to low levels of bacteria, undetectable by traditional methods, and specific to targeted organisms [21]. SPR biosensors meet this demand by offering a method that is sensitive, specific, and capable of delivering real-time or near real-time results. Despite its extensive use in food safety, SPR has not been previously applied to product production in the food industry. To the best of our knowledge, our study is the first to utilize SPR in research for developing culture media, particularly in the realm of cultured meat.

We used SPR to screen 196 chemicals for their ability to bind to Pax7 and MyoD and confirmed the efficacy of the selected chemicals for HWSC proliferation and differentiation using cell-based assays. According to the SPR results, one Pax7+/MyoD− chemical (berbamine), four Pax7+/MyoD+ chemicals (matrine, parthenolide, rutin, and ursolic acid), and one Pax7−/MyoD+ chemical (adenosine) were found. Using a cell-based assay, it was found that the Pax7+/MyoD− chemicals actually decreased the HWSC proliferation as the concentration increased, while the two Pax7+/MyoD+ chemicals, parthenolide (0.5 and 1 µM) and rutin (100 and 200 µM), increased HWSC proliferation and induced normal differentiation. The Pax7−/MyoD+ chemicals promoted differentiation. Overall, we concluded that parthenolide and rutin can be used as HWSC proliferation promoters, and adenosine can be used as an HWSC differentiation promoter.

The natural product library (Selleck Natural Product Library) used in this study is a collection for high-throughput and high-content screening, ensuring high purity. Since this library provides information on chemical substances such as the molecular weight (MW) and hydrophobicity, it has the advantage of predicting the cell permeability of chemicals, which are prerequisites for binding to proteins present in the cytosol [22]. In the past, it was known that only MW affects the cell membrane permeability of chemicals, but recently, factors such as lipophilicity and hydrogen bond donors/acceptors (HBDs/HBAs) have been shown to have greater effects on permeability than the MW [23,24]. The lower the ALogP, the more likely it is to have sufficient solubility and high membrane permeability [25]. The number of HBDs and HBAs positively impacts the solubility and passive diffusion of a chemical, but an excess can have detrimental effects on membrane partitioning and permeability [26]. An optical chemical efficacy requires a harmonious balance between the lipophilic and hydrophilic properties.

Using SPR to measure protein–ligand interactions, we identified a total of six chemicals in a natural product library that binds to either MyoD and/or Pax7. These chemicals were classified into three categories based on their binding patterns to these two proteins: Pax7+/MyoD−, Pax7+/MyoD+, and Pax7−/MyoD+. Both Pax7 and MyoD are critical factors in myogenesis, but they function at different stages of the process [27]. During the early stages of myogenesis, Pax7 is expressed in proliferating myoblasts, where it promotes cell proliferation and inhibits differentiation [28,29]. Pax7 targets the MyoD promoter during myoblast proliferation and is rapidly lost during myogenesis due to its inhibitory effect on myogenin activation [27,28]. In contrast, MyoD is expressed in differentiating myoblasts and is required for the fusion of myoblasts into myotubes [30]. Therefore, Pax7+/MyoD− chemicals target quiescent SCs and early myoblasts, Pax7+/MyoD+ chemicals target myoblasts, and Pax7−/MyoD+ chemicals target myocytes and myotubes [31].

In addition, cell-based assays were carried out to provide information on whether the six chemicals are capable of activating or inhibiting the protein’s biological function, because SPR cannot determine the ligand’s functional activity in a cellular context. The effects of Pax7+/MyoD− and Pax7+/MyoD+ chemicals on cell proliferation and differentiation were evaluated by treating them during the HWSC proliferation process. Of the five chemicals tested, only two chemicals, including parthenolide (0.5 and 1 µM) and rutin (100 and 200 µM), were found to significantly promote HWSC proliferation and induce normal differentiation. Parthenolide, a sesquiterpene lactone mainly found in the leaves of the medicinal plant, *Tanacetum parthenium*, has been studied for its anti-inflammatory, anti-migraine, anti-cancer, and neuroprotective properties [32,33]. Rutin, a flavonoid chemical present in many plants, such as citrus fruits, buckwheat, and asparagus, has been studied for its potential health benefits, including anti-oxidant, anti-inflammatory, anti-cancer properties [34,35]. However, the effect of each chemical on HWSCs or SCs has not been studied, and only a few studies have been reported on C2C12 cells, a myoblast cell line. Parthenolide has been shown to reduce H_2_O_2_-induced cytotoxicity and inhibit the apoptosis of C2C12 cells at 5 μM [33]. Rutin has been shown to attenuate the inflammatory effect induced by lipopolysaccharide in C2C12 cells [36] and prevent muscle atrophy without cytotoxicity by increasing the expressions of MyoD, MyoG, and myosin heavy chain mRNA [37]. And adenosine, a Pax7−/MyoD+ chemical, was observed to significantly enhance HWSC differentiation. The effect of adenosine on SCs or C2C12 cells is also unknown. However, adenosine functions to inhibit cellular metabolism, so it can prevent C2C12 cell death for 20 days under hypoxia conditions [38]. It has been reported that adenosine can interfere with the differentiation, maturation, and activity of dendritic cells through adenosine receptors [39], but in this study, by using muscle SCs, it was found to promote differentiation. This study was conducted to find substances that promote HWSC proliferation and differentiation, and further studies are needed to clarify the effects of parthenolide, rutin, and adenosine on HWSCs.

## 5. Conclusions

The development of cultured meat offers a promising alternative to traditional meat production, presenting potential benefits for environmental sustainability, food security, and human health. As the technology advances, enhancing scalability and cost-efficiency, it can play a significant role in creating a more sustainable and resilient food system. The success of cultured meat development for food sustainability largely depends on the competitiveness in pricing and the edibility of necessary materials like culture media. To discover substances that promote the proliferation of muscle SCs, a general SPR method using a protein–ligand interaction was selected. Although not much has been attempted yet, the high-throughput screening capability of SPR is well suited for the rapidly evolving cultivated meat industry, aiding in the expedited discovery of alternative culture media. The developed method immobilized Pax7 and MyoD, which are specific for the proliferation and differentiation of muscle SCs, as target proteins on a general CM5 chip, and successfully employed natural product chemicals as screening materials. A total of 6 of the 196 chemicals were selected in just two days using a single CM5 chip, and their efficacy was further validated through cell-based assays (proliferation and differentiation), leading to the successful selection of two substances (parthenolide and rutin) that enhance the proliferation of HWSCs and one (adenosine) that promotes the differentiation of HWSCs. Parthenolide and rutin are inexpensive substances that exist in nature and promote the proliferation and differentiation of HWSCs at specific concentrations. That is, both compounds are considered additive candidates for the manufacture of cultured meat media. Although the ultimate goal of cultured meat is to create a medium that can maintain proliferation efficiency even when FBS is completely excluded, the results of this study suggest the possibility that the solution may be found in a natural product.

Additionally, the research method utilized in this study can aid in the discovery of new culture media for cultivated meat production by employing different target proteins. While SPR-based sensors have predominantly been used for detecting various biomarkers that are crucial in the early diagnosis and monitoring of diseases, this study proposes their application in the food sector. Specifically, by targeting membrane proteins that promote cell proliferation, we can identify binding agents that can be developed into media for satellite cell culture. This approach is not limited to cultured meat, but holds potential for broad applications in food-related research, particularly in the functional food sector, due to its adaptability to various cell types.

## Data Availability

The data that support the findings of this study are available from the corresponding author upon request.

## Supplementary Materials

The following supporting information can be downloaded at https://www.mdpi.com/article/10.3390/bios14010024/s1, Table S1: List of 196 chemicals used for ligand screening by SPR.
