# Supplementary material for: Discovery of Novel Stimulators of Pax7 and/or MyoD: Enhancing the Efficacy of Cultured Meat Production through Culture Media Enrichment
Source: Biosensors (Basel). 2023 Dec 30;14(1):24. doi: 10.3390/bios14010024 (PMC10813534; doi:10.3390/bios14010024)
Supplement: Supplementary file 1 [file biosensors-14-00024-s001.zip › biosensors-2675283-supplementary.pdf]

Supplementary Material

Supplementary Table S1. List of 196 chemicals used for ligand screening by SPR

No	Name	MW	Final concentration (μM)	ALogP	HBA_Count	HBD_Count
1	(-)-Epigallocatechin gallate	458.37	157	3.097	3	8
2	(-)-Parthenolide	248.32	197	2.923	3	0
3	(+)-Matrine	248.36	197	1.417	1	0
4	(+)-Usniacin	344.32	12	1.087	4	3
5	(+,-)-Octopamine hydrochloride	189.64	200	0.565	0	3
6	(20S)-Protopanaxatriol	476.73	199	4.62	0	3
7	10,11-Dimethoxystrychnine	394.46	198	1.112	4	0
8	2,6-Dihydroxypurine	152.11	13	-0.718	3	3
9	20-Hydroxyecdysone	480.63	200	1.137	1	3
10	2'-Deoxyinosine	252.23	95	-1.482	4	3
11	2-Methoxy-1,4-naphthoquinone	188.18	197	1.445	3	0
12	4-Demethylepipodophyllotoxin	400.38	200	1.886	6	2
13	4-Hydroxybenzoic acid	138.12	195	1.217	1	1
14	4-Methylumbelliferone	176.17	199	2.104	2	1
15	5-Aminolevulinic acid hydrochloride	167.59	203	-3.318	2	1
16	5-hydroxytryptophan	220.22	200	-1.554	1	3
17	6-Hydroxyflavone	238.24	197	2.894	2	1
18	Adenosine	267.24	45	-1.881	4	4
19	Alizarin	240.21	200	2.324	2	2
20	Aloe-emodin	270.24	11	1.719	2	3
21	Aloin	418.39	198	-0.404	2	7
22	Aminophylline	420.43	126	-2.089	6	4
23	Ammonium glycyrrhizinate	839.96	200	-0.915	8	5
24	Amphotericin B	924.08	24	-2.617	6	10
25	Andrographolide	350.45	200	2.056	2	3
26	Apigenin	270.24	200	2.41	2	3
27	Apocynin	166.17	199	1.311	2	1
28	Arbutin	272.25	202	-0.584	2	5
29	Arecoline hydrobromide	236.11	199	1.234	2	0
30	Artemether	298.37	201	2.029	5	0
31	Artemisinin	282.33	202	1.998	5	0
32	Artesunate	384.42	200	1.845	7	0
33	Asaraldehyde	196.2	199	1.54	4	0
34	Asiatic Acid	488.7	198	4.435	1	3
35	Asiaticoside	959.12	104	-0.101	7	12
36	Astragaloside A	784.97	127	-0.348	5	8
37	Baicalein	270.24	200	2.41	2	3
38	Baicalin	446.36	199	0.608	5	5
39	Berbamine	681.65	147	7.756	5	1
40	Berberine chloride	371.81	108	1.289	4	0
41	Bergenin	328.27	201	-0.849	4	5
42	Betaine	117.15	196	-3.028	0	0
43	Betulinic acid	456.7	44	6.546	1	1
44	Bilobalide	326.3	199	-0.641	6	1
45	Biochanin A	284.26	201	2.366	3	2
46	Biotin	244.31	201	0.67	2	2
47	Borneol	154.25	194	1.975	0	1

48	Caffeic acid	180.16	200	1.443	1	2
49	Catharanthine	336.43	199	3.877	2	1
50	Celastrol	450.61	200	5.475	2	1
51	Cephalomannine	831.9	120	2.937	11	3
52	Chenodeoxycholic acid	392.57	201	4.014	1	2
53	Chloramphenicol	323.13	201	1.025	1	3
54	Cholic acid	408.57	198	2.912	1	3
55	Chrysin	254.24	201	2.652	2	2
56	Chrysophanic acid	254.24	20	2.81	2	2
57	Cinchonidine	294.39	200	2.75	1	1
58	Cinchonine	294.4	31	2.75	1	1
59	Cinnamic acid	148.16	196	1.927	1	0
60	Cordycepin	251.24	199	-1.308	4	3
61	Cortisone acetate	402.48	17	1.619	5	0
62	Cryptotanshinone	296.36	17	3.761	3	0
63	Curcumol	236.35	199	2.786	1	0
64	Cyclosporin A	1202.61	83	4.333	11	5
65	Daidzein	254.24	201	2.382	2	2
66	Daidzin	416.38	199	0.452	4	5
67	Daphnetin	178.14	196	1.415	2	2
68	Daunorubicin hydrochloride	563.98	177	1.01	6	4
69	Dehydrocostus Lactone	230.3	200	3.282	2	0
70	Dehydroepiandrosterone	288.43	198	3.338	1	1
71	Demethylzeylasteral	480.59	200	5.357	3	2
72	Diammonium glycyrrhizinate	856.99	117	-4.247	8	5
73	Dihydromyricetin	320.25	200	1.237	2	6
74	Dihydrothymine	128.13	195	-0.45	2	2
75	D-Mannitol	182.17	198	-2.941	0	6
76	Dopamine hydrochloride	189.64	200	1.155	0	3
77	Doxorubicin (Adriamycin) hydrochloride	579.98	172	0.338	6	5
78	E-Cardamoni	60.06	200	-1.043	1	2
79	Echinacoside	786.73	127	-1.263	8	12
80	Emodin	270.24	200	2.568	2	3
81	Enoxolone	470.68	200	5.656	2	1
82	Epiandrosterone	290.44	100	3.588	1	1
83	Epinephrine bitartrate	333.29	201	-3.996	2	6
84	Esculin	340.28	200	-0.514	4	5
85	Estradiol	272.38	198	3.838	0	2
86	Estriol	288.39	198	2.871	0	3
87	Ferulic acid	194.19	201	1.669	2	1
88	Fisetin	286.24	199	1.872	2	4
89	Flavanone	224.25	196	3.099	2	0
90	Formononetin	268.26	201	2.608	3	1
91	Forskolin	410.5	200	0.837	4	2
92	Gambogic acid	628.75	159	6.957	6	1
93	Genipin	226.23	199	-0.319	3	1
94	Geniposide	388.37	201	-2.066	5	5
95	Geniposidic acid	374.34	43	-2.291	4	5
96	Genistein	270.24	200	2.14	2	3
97	Genistin	432.37	199	0.21	4	6

98	Gentiopicroside	356.32	199	-1.523	5	4
99	Ginkgolide B	424.4	200	-0.854	7	2
100	Ginkgolide C	440.4	200	-1.753	7	3
101	Glabridin	324.37	197	3.999	2	2
102	Glucosamine hydrochloride	215.63	19	-2.422	1	4
103	gossypol-acetic acid	578.61	173	6.388	3	6
104	Gramine	174.24	201	2.197	0	1
105	Guanosine	283.24	198	-2.38	4	5
106	Gynostemma extract	917.13	206	1.059	6	11
107	Hematoxylin	302.28	202	1.691	1	4
108	Hesperetin	302.27	198	2.357	3	3
109	Hesperidin	610.56	164	-0.431	7	8
110	Honokiol	266.334	199	4.88	0	2
111	Hordenine	165.23	200	1.982	0	1
112	Hydrocortisone	362.46	201	1.283	2	2
113	Hyodeoxycholic acid	392.57	199	4.014	1	2
114	Icariin	676.66	74	0.983	7	8
115	Inosine	268.23	198	-2.249	4	4
116	Isoliquiritigenin	256.25	47	2.975	1	3
117	JNJ-1661010	365.45	99	3.271	3	1
118	Kaempferol	286.23	199	1.872	2	4
119	Kitasamycin	785.96	127	2.578	10	3
120	Lactulose	342.3	199	-4.621	3	8
121	L-adrenaline	183.2	22	0.372	0	4
122	L-thyroxine	776.87	129	2.026	2	2
123	Luteolin	286.24	199	2.168	2	4
124	Melatonin	232.28	202	1.555	2	2
125	Methyl gallate	100.12	360	0.959	2	3
126	Methyl vanillate	182.17	198	1.426	3	1
127	Methylmalonate	118.09	195	0.068	2	0
128	Morin hydrate	320.25	200	1.423	2	5
129	Mycophenolic acid	320.34	200	3.157	4	1
130	Myricetin	318.24	198	1.388	2	6
131	Myricitrin	464.38	200	0.347	4	8
132	Naringenin	272.25	198	2.373	2	3
133	Natamycin	665.73	11	-2.824	7	5
134	Neomangiferin	584.48	171	-2.325	5	11
135	Nicotinic acid	123.11	203	0.309	2	0
136	Nobiletin	402.39	201	3.038	8	0
137	Nocodazole	301.32	23	3.008	4	2
138	Norcantharidin	168.15	196	-0.117	4	0
139	Novobiocin sodium	634.61	158	2.747	8	4
140	Oleanolic acid	456.7	46	6.282	1	1
141	Oxytetracycline	460.43	200	-1.904	3	5
142	Paclitaxel	853.91	200	3.055	11	3
143	Paeoniflorin	480.46	200	-1.283	6	4
144	Palmatine	352.4	199	4.161	4	0
145	Palmatine chloride	387.86	199	1.488	4	0
146	Palmitic acid	256.42	199	6.393	1	0
147	Patchouli alcohol	222.37	198	3.427	0	0

148	Phloretic acid	166.17	199	1.708	1	1
149	Piceatannol	244.24	197	2.848	0	4
150	Picroside I	492.47	199	-1.07	6	5
151	Picroside II	512.46	195	-1.796	7	6
152	Piperine	285.34	200	2.864	3	0
153	Polydatin	390.38	200	1.16	2	6
154	Progesterone	314.46	70	3.86	2	0
155	Quercetin	302.24	202	1.63	2	5
156	Quercetin dihydrate	338.27	198	1.216	2	5
157	Quinidine sulfate	648.83	154	4.446	4	2
158	Rapamycin	914.18	22	6.295	10	2
159	Rebaudioside A	967.01	41	-3.476	9	14
160	Reserpine	608.68	21	4.242	9	1
161	Resveratrol	228.24	197	3.09	0	3
162	Rosmarinic acid	360.31	200	2.706	3	4
163	Rotundine	355.43	23	3.599	4	0
164	Rutin	610.52	164	-1.158	6	10
165	Salidroside	300.3	200	-0.443	2	5
166	Schisandrin B	400.46	200	5.067	6	0
167	Scopolamine hydrobromide	384.26	198	1.314	3	1
168	Secoisolariciresinol diglucoside	686.7	146	-0.651	6	10
169	Silymarin	482.44	199	2.592	5	5
170	Sodium danshensu	220.15	32	-0.795	0	3
171	Sophocarpine	246.35	199	1.394	1	0
172	Sophoricoside	432.38	199	0.21	4	6
173	Sorbitol	182.17	198	-2.941	0	6
174	Spectinomycin dihydrochloride	405.27	200	-1.566	4	4
175	Spermidine trihydrochloride	254.63	118	-0.024	0	3
176	Stachydrine	143.18	196	-2.431	0	0
177	Streptozotocin	265.22	200	-2.285	3	4
178	Succinic acid	118.09	195	-0.36	2	0
179	Synephrine hydrochloride	203.67	69	0.997	0	3
180	Syringic acid	198.17	197	1.184	3	1
181	Tanshinone IIA sulfonate	396.39	199	1.873	3	0
182	Tauroursodeoxycholic acid	499.7	198	2.997	1	3
183	Tetracycline hydrochloride	480.9	200	-0.513	3	4
184	Tiglic acid	100.12	200	1.341	1	0
185	Tretinoin	300.4	200	5.551	1	0
186	Tryptamine	160.22	200	1.55	0	2
187	Tyrosol	138.16	195	1.304	0	2
188	Umbelliferone	162.14	197	1.657	2	1
189	Ursodiol	392.57	201	4.014	1	2
190	Ursolic acid	456.7	199	6.327	1	1
191	Vanillin	152.15	197	1.33	2	1
192	Vanillylacetone	194.23	196	1.792	2	1
193	Vincristine sulfate	923.04	108	3.365	8	1
194	Vitamin C	176.12	199	-1.709	2	4
195	Xanthone	196.2	199	2.964	2	0
196	Yohimbine hydrochloride	390.9	31	3.204	2	2
